# Supplementary material for: Comparison of the Morphological Characteristics of South African Sub-Elite Female Football Players According to Playing Position
Source: Int J Environ Res Public Health. 2021 Mar 31;18(7):3603. doi: 10.3390/ijerph18073603 (PMC8036279; doi:10.3390/ijerph18073603)
Supplement: Supplementary file 1 [file ijerph-18-03603-s001.pdf]

THE HEATH-CARTER ANTHROPOMETRIC SOMATOTYPE

- INSTRUCTION MANUAL -

J.E.L. Carter, Ph.D.
Department of Exercise and Nutritional Sciences
San Diego State University
San Diego, CA. 92182-7251. U.S.A.
Email: lindsay.carter@sdsu.edu

The figures in this manual may be reproduced for class use
without specific permission.

Revised by J.E.L. Carter
San Diego, CA. U.S.A.

This revision is adapted from the original instruction manual by the author and a later version published in a CD-Rom titled “Anthropometry Illustrated” (Ross, Carr & Carter, 1999), in association with TeP and ROSSCRAFT,
Surrey, Canada.

March 2002

Part 1: The Heath-Carter Anthropometric Somatotype

# - Instruction Manual -

J.E.L. Carter, Ph.D.
Department of Exercise and Nutritional Sciences
San Diego State University
San Diego, CA. 92182-7251. U.S.A.

Introduction

The technique of somatotyping is used to appraise body shape and composition. The somatotype is defined as the quantification of the present shape and composition of the human body. It is expressed in a three-number rating representing endomorphy, mesomorphy and ectomorphy components respectively, always in the same order. Endomorphy is the relative fatness, mesomorphy is the relative musculo-skeletal robustness, and ectomorphy is the relative linearity or slenderness of a physique. For example, a 3-5-2 rating is recorded in this manner and is read as three, five, two. These numbers give the magnitude of each of the three components. Ratings on each component of ½ to 2½ are considered low, 3 to 5 are moderate, 5½ to 7 are high, and 7½ and above are very high (Carter & Heath, 1990). The rating is phenotypical, based on the concept of geometrical size-dissociation and applicable to both genders from childhood to old age.

The Heath-Carter method of somatotyping is the most commonly used today. There are three ways of obtaining the somatotype.

(1) The *anthropometric* method, in which anthropometry is used to estimate the criterion somatotype.

(2) The *photoscopic* method, in which ratings are made from a standardized photograph.

(3) The *anthropometric plus photoscopic* method, which combines anthropometry and ratings from a photograph - it is the criterion method.

Because most people do not get the opportunity to become criterion raters using photographs, the anthropometric method has proven to be the most useful for a wide variety of applications.

Purpose

The purpose of this chapter is to provide a simple description of the anthropometric somatotype method. It is intended for those who are interested in learning "how to do it". To obtain a fuller understanding of somatotyping, its uses and limitations, the reader should consult "Somatotyping - Development and Applications", by Carter and Heath (1990).

The Anthropometric Somatotype Method

Equipment for anthropometry

Anthropometric equipment includes a stadiometer or height scale and headboard, weighing scale, small sliding caliper, a flexible steel or fiberglass tape measure, and a skinfold caliper. The small sliding caliper is a modification of a standard anthropometric caliper or engineer's vernier type caliper. For accurate measuring of biepicondylar breadths the caliper branches must extend to 10 cm and the tips should be 1.5 cm in diameter (Carter, 1980). Skinfold calipers should have upscale interjaw

pressures of 10 gm/mm^2 over the full range of openings. The Harpenden and Holtain calipers are highly recommended. The Slim Guide caliper produces almost identical results and is less expensive. Lange and Lafayette calipers also may be used but tend to produce higher readings than the other calipers (Schmidt & Carter, 1990). Recommended equipment may be purchased as a kit (TOM Kit) from Rosscraft, Surrey, Canada (email: rosscraft@shaw.ca, or www.tep2000.com).

Measurement techniques

Ten anthropometric dimensions are needed to calculate the anthropometric somatotype: stretch stature, body mass, four skinfolds (triceps, subscapular, supraspinale, medial calf), two bone breadths (biepicondylar humerus and femur), and two limb girths (arm flexed and tensed, calf). The following descriptions are adapted from Carter and Heath (1990). Further details are given in Ross and Marfell-Jones (1991), Carter (1996), Ross, Carr and Carter (1999), Duquet and Carter (2001) and the ISAK Manual (2001).

Stature (height). Taken against a height scale or stadiometer. Take height with the subject standing straight, against an upright wall or stadiometer, touching the wall with heels, buttocks and back. Orient the head in the Frankfort plane (the upper border of the ear opening and the lower border of the eye socket on a horizontal line), and the heels together. Instruct the subject to stretch upward and to take and hold a full breath. Lower the headboard until it firmly touches the vertex.

Body mass (weight). The subject, wearing minimal clothing, stands in the center of the scale platform. Record weight to the nearest tenth of a kilogram. A correction is made for clothing so that nude weight is used in subsequent calculations.

Skinfolds. Raise a fold of skin and subcutaneous tissue firmly between thumb and forefinger of the left hand and away from the underlying muscle at the marked site. Apply the edge of the plates on the caliper branches 1 cm below the fingers of the left hand and allow them to exert their full pressure before reading at 2 sec the thickness of the fold. Take all skinfolds on the right side of the body. The subject stands relaxed, except for the calf skinfold, which is taken with the subject seated.

Triceps skinfold. With the subject's arm hanging loosely in the anatomical position, raise a fold at the back of the arm at a level halfway on a line connecting the acromion and the olecranon processes.

Subscapular skinfold. Raise the subscapular skinfold on a line from the inferior angle of the scapula in a direction that is obliquely downwards and laterally at 45 degrees.

Supraspinale skinfold. Raise the fold 5-7 cm (depending on the size of the subject) above the anterior superior iliac spine on a line to the anterior axillary border and on a diagonal line going downwards and medially at 45 degrees. (This skinfold was formerly called suprailiac, or anterior suprailiac. The name has been changed to distinguish it from other skinfolds called "suprailiac", but taken at different locations.)

Medial calf skinfold. Raise a vertical skinfold on the medial side of the leg, at the level of the maximum girth of the calf.

Biepicondylar breadth of the humerus, right. The width between the medial and lateral epicondyles of the humerus, with the shoulder and elbow flexed to 90 degrees. Apply the caliper at an angle approximately bisecting the angle of the elbow. Place firm pressure on the crossbars in order to compress the subcutaneous tissue.

Biepicondylar breadth of the femur, right. Seat the subject with knee bent at a right angle. Measure the greatest distance between the lateral and medial epicondyles of the femur with firm pressure on the crossbars in order to compress the subcutaneous tissue.

Upper arm girth, elbow flexed and tensed, right. The subject flexes the shoulder to 90 degrees and the elbow to 45 degrees, clenches the hand, and maximally contracts the elbow flexors and extensors. Take the measurement at the greatest girth of the arm.

Calf girth, right. The subject stands with feet slightly apart. Place the tape around the calf and measure the maximum circumference.

Read stature and girths to the nearest mm, biepicondylar diameters to the nearest 0.5 mm, and skinfolds to the nearest 0.1 mm (Harpenden caliper) or 0.5 mm on other calipers.

Traditionally, for the anthropometric somatotype, the larger of the right and left breadths and girths have been used. When possible this should be done for individual assessment. However, in large surveys it is recommended that all measures (including skinfolds) be taken on the right side. The anthropometrist should mark the sites and repeat the complete sequence a second time. For further calculations, the duplicated measurements should be averaged. For more reliable values, relatively inexperienced measurers should take triplicate measurements and use the median value.

Reliability of measurements

The advantages of anthropometry are lost unless the measurements are accurate and reliable (i.e. precise). It is essential to learn precise measurement techniques and accurate calculations. Although at first sight anthropometry appears easy to the beginning investigator, obtaining a high level of skill and reliability requires training under a criterion anthropometrist and considerable practice.

Although calculation of the Heath-Carter anthropometric somatotype is an objective procedure, the validity of the rating depends on the reliability of the measurements used. Investigators should report test-retest reliability of the measurements. In comparisons of distributions of two independent measures on the same subjects, the means should not differ significantly, and the Pearson product-moment r should be above 0.90. Specifically, height and weight should have test-retest values of r = 0.98. Girths and diameters should have r's between 0.92 and 0.98. For skinfolds r's between 0.90 and 0.98 are reasonable. Currently, many anthropometrists use the technical error of measurement (TEM) for evaluating the consistency, or precision, of the measurer on a given variable. The TEM is the square root of the sum of the differences between measures one and two squared, divided by twice the number of subjects (Cameron, 1984, Norton and Olds, 1996). The TEM provides an estimate of the measurement error that is in the units of measurement of the variable. This value indicates that two thirds of the time a measurement should come within +/- of the TEM. The TEM can be converted to a percentage of the mean of the total number of measures (grand mean). This allows for comparisons among measures or for groups of variables. Generally, the TEM for skinfolds should be about 5%, and that for breadths and girths 1%, and for height about 0.5% The equations are as follows:

$$\mathbf{TEM = (\sum d^2 / 2n)^{0.5}\ ;\ and\ \%TEM = 100\ (TEM/grand\ mean)}$$

Calculating the Anthropometric Somatotype

There are two ways to calculate the anthropometric somatotype.

(A) Enter the data onto a somatotype rating form.

(B) Enter the data into equations derived from the rating form.

The use of the rating form will be described first. Figures 1 and 2 are examples of calculations using the rating form. It is assumed that the measurements have been recorded on an appropriate

recording form and average or median values calculated before transfer to the rating form. A blank rating form is provided in Figure 3.

A. The Heath-Carter Somatotype Rating Form

(1) Record pertinent identification data in top section of rating form.

Endomorphy rating (steps 2-5)

(2) Record the measurements for each of the four skinfolds.

(3) Sum the triceps, subscapular, and supraspinale skinfolds; record the sum in the box opposite SUM3 SKINFOLDS. Correct for height by multiplying this sum by (170.18/height in cm).

(4) Circle the closest value in the SUM3 SKINFOLDS table to the right. The table is read vertically from low to high in columns and horizontally from left to right in rows. "Lower limit" and "upper limit" on the rows provide exact boundaries for each column. These values are circled only when SUM3 SKINFOLDS are within 1 mm of the limit. In most cases circle the value in the row "midpoint".

(5) In the row for endomorphy circle the value directly under the column for the value circled in number (4) above.

Mesomorphy rating (steps 6-10)

(6) Record height and breadths of humerus and femur in the appropriate boxes. Make the corrections for skinfolds before recording girths of biceps and calf. (Skinfold correction: Convert triceps skinfold to cm by dividing by 10. Subtract converted triceps skinfold from the biceps girth. Convert calf skinfold to cm, subtract from calf girth.)

(7) In the height row directly to the right of the recorded value, circle the height value nearest to the measured height of the subject. (Note: Regard the height row as a continuous scale.)

(8) For each bone breadth and girth circle the number nearest the measured value in the appropriate row. (Note: Circle the lower value if the measurement falls midway between two values. This conservative procedure is used because the largest girths and breadths are recorded.)

(9) Deal only with columns, not numerical values for the two procedures below. Find the average deviation of the circled values for breadths and girths from the circled value in the height column as follows:

> (a) Column deviations to the right of the height column are positive deviations. Deviations to the left are negative deviations. (Circled values directly under the height column have deviations of zero and are ignored.)
>
> (b) Calculate the algebraic sum of the ± deviations (D). Use this formula: mesomorphy = (D/8) + 4.0. Round the obtained value of mesomorphy to the nearest one-half (½) rating unit.

(10) In the row for mesomorphy circle the closest value for mesomorphy obtained in number 9 above. (If the point is exactly midway between two rating points, circle the value closest to 4 in the row. This conservative regression toward 4 guards against spuriously extreme ratings.)

Ectomorphy rating (steps 11-14).

(11) Record weight (kg).

(12) Obtain height divided by cube root of weight (HWR). Record HWR in the appropriate box.

(13) Circle the closest value in the HWR table to the right. (See note in number (4) above.)

(14) In the row for ectomorphy circle the ectomorphy value directly below the circled HWR.

(15) Move to the bottom section of the rating form. In the row for Anthropometric Somatotype, record the circled ratings for Endomorphy, Mesomorphy and Ectomorphy.

(16) Sign your name to the right of the recorded rating.

The identification data in the upper section of the rating form are somewhat arbitrary. Investigators may change these to suit their purposes.

Principles of the calculations

Two principles are important in understanding the calculation of mesomorphy on the rating form. (1) When the measurements of bone breadths and limb girths lie to the right of the circled height column, the subject has greater musculo-skeletal robustness relative to height (i.e. higher mesomorphy) than a subject whose values lie to the left of the height column. The average deviation of the circled values for breadths and girths is the best index of average musculo-skeletal development relative to height. (2) The table is constructed so that the subject is rated 4 in mesomorphy when the average deviation falls in the column under the subject's height, or when the four circled values fall in the subject's height column. That is, the average deviation (±) to the left or right of the height column is added to or subtracted from 4.0 in mesomorphy.

Height-Weight ratio calculation

The height-weight ratio (HWR), or height divided by the cube root of weight (stature/mass$^{1/3}$) as it is used in somatotyping, may be determined by using a hand calculator. A calculator with a y to the x power (y^x) key is needed. To get the cube root, enter mass, i.e. base (y), press y^x, enter .3333, and press 'equals'. If there is an INV y^x function, this may be used instead by entering 3 (for the cube root).

Limitations of the rating form

Although the rating form provides a simple method of calculating the anthropometric somatotype, especially in the field, it has some limitations. First, the mesomorphy table at the low and high ends does not include some values for small subjects, e.g. children, or for large subjects, e.g. heavy weightlifters. The mesomorphy table can be extrapolated at the lower and upper ends for these subjects. Second, some rounding errors may occur in calculating the mesomorphy rating, because the subject's height often is not the same as the column height. If the anthropometric somatotype is regarded as an estimate this second limitation is not a serious problem. Nevertheless, the following procedures described in Carter (1980) and Carter and Heath (1990) can correct these problems.

B. Equations for a decimal anthropometric somatotype

The second method of obtaining the anthropometric somatotype is by means of equations into which the data are entered.

endomorphy $= -0.7182 + 0.1451 (X) - 0.00068 (X^2) + 0.0000014 (X^3)$

where X = (sum of triceps, subscapular and supraspinale skinfolds) multiplied by (170.18/height in cm). This is called height-corrected endomorphy and is the preferred method for calculating endomorphy.

The equation to calculate mesomorphy is:

mesomorphy = 0.858 x humerus breadth + 0.601 x femur breadth + 0.188 x corrected arm girth + 0.161 x corrected calf girth – height 0.131 + 4.5.

Three different equations are used to calculate ectomorphy according to the height-weight ratio:

If HWR is greater than or equal to 40.75 then
ectomorphy = 0.732 HWR - 28.58

If HWR is less than 40.75 but greater than 38.25 then
ectomorphy = 0.463 HWR - 17.63

If HWR is equal to or less than 38.25 then
ectomorphy = 0.1

For subjects 573 and B-188 respectively (Figures 1, 2), the resulting somatotypes (using height corrected endomorphy) are 1.6-5.4-3.2, and 3.0-2.1-4.8,

The preceding equations, derived from data used by Heath and Carter (1967), use metric units. The equation for endomorphy is a third degree polynomial. The equations for mesomorphy and ectomorphy are linear. (When the HWR is below 40.75 a different equation is used for ectomorphy.) If the equation calculation for any component is zero or negative, a value of 0.1 is assigned as the component rating, because by definition ratings cannot be zero or negative.

Checking the results

Now that the anthropometric somatotype has been calculated, is the result logical? There are several ways to check your results for measurement or calculation errors.

Using the rating form examples in Figures 1 and 2, the resulting somatotypes rounded to the nearest half-unit, are 1½-5½-3 and 3-2-5 for subjects 573 and B-188 respectively. Are these reasonable somatotypes? Certain somatotype ratings are not biologically possible, although our examples are not among them. For example, a 2-2-2 or a 7-8-7 are impossible somatotypes. Generally, somatotypes high in endomorphy and/or mesomorphy cannot also be high in ectomorphy. Conversely, those high in ectomorphy cannot be high in endomorphy and/or mesomorphy; and those low in endomorphy and mesomorphy must be high in ectomorphy.

Next, look at the pattern of circled values in the endomorphy and mesomorphy sections of the rating form. Are there inconsistencies in the data? For endomorphy, are the skinfold values reasonable? For mesomorphy, is there one measure (excluding height) that is quite far apart from the others? In Figure 1, upper limb circled values are slightly to the right of, and larger relative to height, than the lower limb measures. However, this pattern is not unusual and is quite acceptable in this case. On the other hand, if the femur width was 7.95 cm instead of 9.75 cm, or corrected calf girth was 44.9 cm instead of 37.1 cm, such large deviations would suggest errors. Check for errors in recording and remeasure the subject if possible. Also, check to see that the correct skinfolds in cm have been subtracted for the corrected girth values. In Figure 2, the small corrected biceps girth (23.4 cm) looks suspiciously low, but in this subject it truly represented her small muscular development in the upper limb.

If the calculation for any component is zero or negative, a value of 0.1 is assigned as the component rating, because by definition ratings cannot be zero or negative. The photoscopic rating would be one-half (½). If such low values occur the raw data should be checked. Values less than 1.0 are highly unlikely to occur for endomorphy and mesomorphy, but are not unusual for ectomorphy. Component ratings should be rounded to nearest 0.1 of a unit, or nearest half-unit depending on their subsequent use.

After the values are entered into the equations (either by calculator or computer program) rather than onto the rating form, it is impossible to check the pattern of values in either the endomorphy or mesomorphy section as in the rating form, although the raw values can be examined for errors. This is a limitation of using the equations. Further checking can be done for either method by using the HWR and by plotting the somatotype.

There is a relationship between the HWR and the likely somatotypes (see Fig. 4). The somatotypes in the rows are those most likely to occur for the given HWR. For example, given a HWR of approximately 49.6, the most likely somatotypes are 1-1-8, 1-2-9 or 2-1-9. (The hyphens are left out of the somatotypes to conserve space.) The next most likely somatotypes are those in the rows directly above and below the row for 49.6. If none of these somatotypes match or are not close when interpolating for half-unit ratings, there may be errors in the data or calculations. However, other factors such as heavy meals or dehydration can affect body weight sufficiently to alter the "normal" HWR.

For subject 573, HWR = 43.4, and Figure 4 shows that in the row for a HWR of 43.64 the somatotypes 1-6-3 and 2-5-3 occur. His 1½-5½-3 is a combination of these two ratings, therefore his anthropometric rating agrees with that expected from the HWR table. For subject B-188, HWR = 45.6, her 3-2-5 somatotype appears in the row above that for her HWR. Her ectomorphy is borderline between 4½ and 5, which suggests that she might be a 3-2-4½, i.e. half way between the two rows. The somatotypes for both subjects appear to be reasonable.

[A program for calculating individual and group data is provided in a Microsoft Excel© program, HC-Stype Calculator.xls. See Part 3.]

Plotting the Somatotype

Traditionally, the three-number somatotype rating is plotted on a two-dimensional somatochart using X,Y coordinates derived from the rating (see Fig. 4). The coordinates are calculated as follows:

X = ectomorphy - endomorphy
Y = 2 x mesomorphy - (endomorphy + ectomorphy)

For subject 573, X = 1.5, and Y = 6.5. For subject B-188, X = 2.0, and Y = -4.0. These points on the somatochart are called somatoplots. If the somatoplot for the subject is far from that expected when compared to a suitable reference group, check the data and calculations. Because Figure 5 is quite crowded with numbers, the final somatoplots could be projected onto a somatochart without the numbers. Figures 6a,b are two blank somatocharts, one with printed somatotypes and one without. These may be copied for use.

Other versions of the rating form may be used instead. See Fig. 3, p. 153 in Carter (1996) for the adaptation by Tim Olds. For descriptions and illustrations in Spanish of the anthropometric somatotype rating form and somatocharts refer to the web site of Mariano A. Godnic (ARG), www.nutrinfo.com.ar.

The Somatotype Photograph

The somatotype photograph is a valuable record of the physique, especially when change is expected and for longitudinal growth studies. It can be used as a supplement to the anthropometric somatotype rating, in assessment of body image, and in association with the anthropometric profile. Even if you are not a qualified somatotype rater, you can look for the correspondence between the anthropometric somatotype and what you see in the photograph. In other words, the photo provides you with the visual image of what a particular 2-5-3 or 6-3-1 looks like. Details of how to rate the photographs, with examples, are described in Carter and Heath (1990, App. I).

The somatotype photograph requires standardized poses of front, side and rear views of the subject. The recommended minimal equipment consists of a good quality 35 mm camera, with an 80 mm lens and attached flash. The camera should be mounted on a tripod, at 5.8 m from the subject and the lens height at approximately mid-stature for most subjects in the sample. Commercially developed standard color print film (ASA 200 or 400) is quite satisfactory and relatively inexpensive. The subject should be dressed in minimal clothing such as light-weight swim suits (single or two-piece), or running shorts. A more permanent somatotype station should include a data board, turntable, standard scale, white background, and flood or flash lights. (See Carter & Heath, 1990, App. I, for other options.) Digital photography is now a viable option if care is taken to keep the focal length long to reduce distortion of the physique.

Somatotype categories

Somatotypes with similar relationships between the dominance of the components are grouped into categories named to reflect these relationships. Figure 7 shows somatotype categories as represented on the somatochart. The definitions are given below. Subject 573 is an ectomorphic mesomorph (or ecto-mesomorph), and subject B-188 is an endomorphic ectomorph (or endo-ectomorph). All other somatotypes plotted within the same area are assigned the same category name. The frequencies of somatotypes within categories (or combined categories) can be used to describe the overall distribution of samples or for comparing them using a Chi-square analysis. The definitions of 13 categories are based on the areas of the 2-D somatochart (Carter and Heath, 1990).

Central: no component differs by more than one unit from the other two.

Balanced endomorph: endomorphy is dominant and mesomorphy and ectomorphy are equal (or do not differ by more than one-half unit).

Mesomorphic endomorph: endomorphy is dominant and mesomorphy is greater than ectomorphy.

Mesomorph-endomorph: endomorphy and mesomorphy are equal (or do not differ by more than one-half unit), and ectomorphy is smaller.

Endomorphic mesomorph: mesomorphy is dominant and endomorphy is greater than ectomorphy.

Balanced mesomorph: mesomorphy is dominant and endomorphy and ectomorphy are equal (or do not differ by more than one-half unit).

Ectomorphic mesomorph: mesomorphy is dominant and ectomorphy is greater than endomorphy.

Mesomorph-ectomorph: mesomorphy and ectomorphy are equal (or do not differ by more than one-half unit), and endomorphy is smaller.

Mesomorphic ectomorph: ectomorphy is dominant and mesomorphy is greater than endomorphy.

Balanced ectomorph: ectomorphy is dominant and endomorphy and mesomorphy are equal (or do not differ by more than one-half unit).

Endomorphic ectomorph: ectomorphy is dominant and endomorphy is greater than mesomorphy.

Endomorph-ectomorph: endomorphy and ectomorphy are equal (or do not differ by more than one-half unit), and mesomorphy is lower.

Ectomorphic endomorph: endomorphy is dominant and ectomorphy is greater than mesomorphy.

The 13 categories can be simplified into four larger categories:

Central: no component differs by more than one unit from the other two.

Endomorph: endomorphy is dominant, mesomorphy and ectomorphy are more than one-half unit lower.

Mesomorph: mesomorphy is dominant, endomorphy and ectomorphy are more than one-half unit lower.

Ectomorph: ectomorphy is dominant, endomorphy and mesomorphy are more than one-half unit lower.

The three-dimensional somatotype

Because the somatotype is a three-number expression meaningful analyses can be conducted only with special techniques. Somatotype data can be analyzed by both traditional and non-traditional descriptive and comparative statistical methods Although descriptive statistics are used for each of the components, comparative statistics should be made in the first instance using the whole (or global) somatotoype rating. This, is followed by analysis of separate components. Some of these analyses are described in Part 2. Here are some useful definitions:

somatopoint (S). A point in three-dimensional space determined from the somatotype which is represented by a triad of x, y and z coordinates for the three components. The scales on the coordinate axes are component units with the hypothetical somatotype 0-0-0 at the origin of the three axes.

somatotype attitudinal distance (SAD). The distance in three dimensions between any two somatopoints. Calculated in component units.

somatotype attitudinal mean (SAM). The average of the SADs of each somatopoint from the mean somatopoint (S) of a sample.

The SAD represents the "true" distance between two somatopoints (A and B). The SAD is calculated as follows:

$$SAD_{A,B} = \sqrt{[(endomorphy_A - endomorphy_B)^2 + (mesomorphy_A - mesomorphy_B)^2 + (ectomorphy_A - ectomorphy_B)^2]}$$

Where A and B are two individuals, two different times for one individual, or two means.

The **SAM** is calculated by dividing the sum of the SADs from their mean somatopoint by the number of subjects.

****

Part 2: Somatotype Analysis

[This article is similar to sections in Ross, WD, Carr, RV and Carter, JEL, (1999). Anthropometry Illustrated (CD-Rom). Surrey, Canada: Turnpike Electronic Publications, Inc., 1999, used with permission.]

The purpose of Part 2 is to draw attention to some basic concepts of somatotype analysis, comment on recent applications, and provide a guide for investigators. The intent is to give general rather than detailed procedures. For the latter the reader is referred to Carter (1996), Carter et al. (1983), Carter and Heath (1990), Duquet and Carter (1996) and Sodhi (1991).

The most widely applied method for obtaining the somatotype is the anthropometric method of Heath and Carter (1967), which has been modified slightly since it was first, published. Important changes include the conversion to a rating form in metric units, the use of a height adjustment for endomorphy which is now standard procedure, and the use of equations, instead of the rating form, to calculate the component ratings (Carter and Heath, 1990). Recently, Rempel (1994) has developed modifications of the rating form and equations so that the scales are truly size dissociated. His modifications are sound and when tested further they will probably be accepted as the preferred methods for calculating somatotype ratings.

Analysis of the three-number somatotype rating presents some unusual problems to the uninitiated. How should such a rating be analyzed? Early solutions typically involved either analyzing the three component ratings separately, or grouping somatotypes and comparing the frequencies. During the 1970s and 1980s more sophisticated analyses, appropriate for analysis of the somatotype as a whole, were developed. These were summarized in a series of publications, Hebbelinck et al. (1973), Duquet and Hebbelinck (1977), Carter (1980), Carter et al. (1983) and Carter and Heath (1990). An important element of these developments was the ability to analyze whole somatotypes in two or three dimensions using appropriate equations, which led to application of conventional statistical analysis. These equations quantified the distances between somatotypes. The two and three-dimensional distances between pairs of somatotypes are called the somatotype dispersion distance (SDD) and somatotype attitudinal distance (SAD) respectively. The average of the distribution of somatotypes about their mean is called the somatotype dispersion mean (SDM) in two dimensions, and somatotype attitudinal mean (SAM) in three dimensions. Because the SAD and SAM, which are based in three dimensions, contain more accurate information about the true distances between somatotypes than the SDD and SDM, which are based in two dimensions, the three dimensional approach is recommended for most analyses. These equations are given in the references cited above, as well as later in Part 3.

Procedures

The recommended procedures for obtaining and analyzing somatotype data are presented below, but all are not appropriate for every study. The listing can be used as a guide. In general, the whole somatotype (i.e. global or Gestalt somatotype) should be analyzed first. If there are differences between groups, analysis of the components should follow.

1. ***Anthropometry***. Obtain the 10 body measures needed for calculating the anthropometric somatotype. Take repeated measures to determine the technical errors of measurement, both for the measurers and for comparisons with a criterion anthropometrist, and report them. Use error detection methods when appropriate.

2. ***Rating form and equations***. Calculate the somatotype by entering the measures onto the rating form, or into the equations. The rating form should be used for beginners, for individual assessment and counseling, and for checking the pattern of measures in unusual somatotypes. Once data are entered into equations some errors are likely to go undetected. However, once proficiency is obtained in measurements, the use of equations in calculators or computers is obviously the most efficient way to handle large collections of data.

3. ***Somatocharts***. Plot individual and mean somatotypes on a somatochart. The somatoplots on the somatochart provide an important visual display, either for individuals or means, and should be utilized routinely. The shape of the distribution of somatoplots of a sample imparts considerable useful information and should be preliminary to, or accompany, statistical analysis. It is vital to interpreting results and should be included in publications when possible.

4. ***Categories***. Determine the frequencies of somatotypes in categories, e.g. balanced ectomorph, endo-mesomorph. When there are low frequencies in adjacent categories on the somatochart they can be combined for a better summary and analysis. Caution must be used in combining categories - they must make biological as well as statistical sense.

5. ***Descriptive statistics***. Calculate and report descriptive statistics for the sample variables, including endomorphy, mesomorphy, ectomorphy, SAM and SDM, as well as age, height, weight, and the height/weight$^{1\backslash 3}$. For statistical purposes, report individual and mean somatotypes to one decimal place, e.g. 2.4-4.1-3.6. but for simplicity in conversation and written summaries round the rating to the nearest half-unit, i.e. 2½-4-3½. Standard deviations can be reported to two decimals. However, do NOT report the standard deviations within the three-number somatotype rating as this destroys the concept of the whole somatotype. For example, try to avoid writing that the somatotype of the groups was 2.4 (±0.5), 4.1 (±0.7), 3.6 (±0.6); in tables put the standard deviation under the mean, not in the next column. Sometimes a certain form might be desirable or required by editors.

6. ***Comparative statistics***. Determine what comparative statistics are needed, e.g. non-parametric and parametric. The choice of procedures will depend on consideration of the usual limitations of subject and sample characteristics, as well as the statistical assumptions and design.

7. ***Non-parametric comparisons***. When the frequency and relative frequency of somatotypes according to categories on the somatochart are calculated, Chi-square or comparative ratio analyses are applicable. In longitudinal studies, the changes in individual and mean somatotypes can be quantified by summing the sequential distances during the time of the study. This is called the migratory distance (MD). Because the MD does not indicate direction, it should be used in conjunction with the somatoplots of the individuals or means. Other techniques such as Intensity Distance, Cutting Line Principle, I-index, and Percent Overlap can also be used but are not often applied. (See Carter and Heath, 1990).

8. Parametric statistics.

(a) ***Computations***. Parametric statistics of the whole somatotype can be used for comparisons among groups. Care must be taken to use the correct computational elements for calculating the variances and mean differences, and this change depending upon whether a t-test or analysis of variance (ANOVA) are used (Carter et al., 1983). Two different statistical tests are applied when comparing two or more groups: (1) tests for differences between somatotype means (S_1 - S_n), and (2) tests for differences in the scatter of somatotypes about their means (SAM_1 - SAM_n). In addition, the ANOVAs for repeated measures are different from those for independent groups. (There is no suitable t-test for repeated measures for the whole somatotype.) Because SADs are simply distances between two points, differences among SAMs can be analyzed by standard statistical tests. The procedures that follow in 8(b,c) refer to analysis of mean somatotypes only.

(b) ***Between-group comparisons***. For differences between mean somatotypes, use the SADs as measurement units (Carter et al., 1983). Specific programs can be written for these analyses, or computational statements can be written into standard statistical programs (e.g. SPSS) or into spreadsheets (e.g. MS Excel) to calculate the correct computational elements. For between-group comparisons, the use of a t-ratio for two groups, or an F-ratio for two or more groups, is appropriate. When the F-ratio is significant for more than two groups use post-hoc tests for differences between means, such as Tukey or Scheffe, which are conservative with respect to type I errors (Winer, 1971). The "effect size" for the differences between means that is considered of practical and statistical significance is SAD = 0.5 (i.e. ½ unit) for pair wise comparisons (Carter & Heath, 1990). In these comparisons the somatotype is regarded as a whole and the degrees of freedom for the F-ratio is based on the usual number of groups (k-1) and number of subjects (n-k)

In proposing an alternative to the methods of Carter et al. (1983) for analyzing somatotypes of independent groups, Cressie et al. (1986) claimed that using the SAD prematurely collapsed the three somatotype component vectors into a scalar SAD value, together with the use of inappropriate degrees of freedom for the one-way ANOVA and F-ratio. They suggest increasing the degrees of freedom to include those for the three separate components, thus increasing the likelihood of type I errors when compared to the method of Carter et al. (1983). They recommended using a one-way MANOVA for analyzing the three components. However, this procedure does NOT analyze the whole somatotype. It

prematurely separates the somatotype into three components. Their premise is false in that it compromises (or denies) the integrity of the whole somatotype and erroneously increases the degrees of freedom. Furthermore, the SAD should be treated as any other derived variable and not be assigned degrees of freedom based on the variables from which it is calculated. The degrees of freedom for a given variable are not normally based on the number of variables, which contribute to them. Examples include the BMI or HWR, or vectors contributing to complex biomechanical, physiological or growth variables. Therefore, why do it for the somatotype? Such a notion violates the basic biological premise of the somatotype as a whole. However, other investigators may wish to apply and evaluate either method of determining the appropriate degrees of freedom. For a more recent discussion of these points, see Carter and Duquet (1998, 1999) and Cressie (1998, 1999). (Other applications of analyses by Cressie et al. are discussed below.)

(c) ***Repeated measures designs***. If a repeated measures design is used, as in growth or training studies, then different equations have to be used for analysis of the somatotype as a whole (Carter et al., 1983). A two-way ANOVA is appropriate for this design, but there is no analogous somatotype t-test for correlated data. If the F-ratios are significant, post-hoc tests are applied as noted above.

(d) ***Analysis of separate components***. If there are no differences among somatotypes by group, usually there is no need for further tests of the separate components. If differences among somatotype means are found using 8 (b,c) above, then the next step is to see which components are responsible for these differences. If considered as a univariate problem, the simplest analysis consists of applying t-tests or F-tests to each component, in either independent or repeated measures designs. An adjustment for the total number of comparisons should be used to reduce the likelihood of Type I errors. Because there are three pairs of comparisons in a comparison of two groups or two occasions, one each for endomorphy, mesomorphy and ectomorphy, the correct experiment wise error rate can be calculated by the Bonferroni technique, in which the alpha level is divided by the number of comparisons.

Because there are three components or variables in the somatotype, a multivariate approach is more powerful. The three components can be examined together for their joint contributions to differences among discrete groups using a discriminant analysis (DA). The dependent variables (i.e. the three components) are used to first select the variables that contribute significantly to group differences, and then these variables are entered into a linear combination to predict the correct group membership, with statistics such as overall F-ratios, Wilks' lambda and canonical correlations as measures of significance. The relative contribution of each component, which enters into the analysis, is calculated. One advantage of DA is that although each component taken separately may not differ among groups, when taken together (i.e. two or three) they could contribute to a reasonable and significant separation into groups. The canonical correlation squared gives an estimate of the variance accounted for, and the percentage of subjects correctly classified is also a useful indicator of differences among groups. (DA can only be used with independent groups.)

As an alternative to DA analysis, Cressie et al. (1986) recommended the use of a one-way MANOVA which uses Wilks' lambda at the test statistic, and if significant, pair wise comparisons are conducted by

using Hotelling's T^2, with a Bonferroni adjusted alpha level. As noted above this is an appropriate analysis for the separate components, but not for the whole somatotype. Beunen et al. (1987), Bell (1994) and Rosique et al. (1994), applied the MANOVA procedures. However, these authors erroneously claim to have analyzed the "whole somatotype", but in fact analyzed the three separate components.

(e) ***Partial correlations***. The stability of each component over time (i.e. repeated measures) can be assessed by calculating the interage partial correlations for one component while holding the other two components constant, i.e. $\mathbf{r_{1,23}}$. Because there are moderate correlations between the somatotype components and there is reduced meaning of component values when taken out of the context of the somatotype, partial correlations are more appropriate and meaningful (Hebbelinck et al., 1995).

Summary. This review has attempted to reinforce some basic principles of somatotype analysis and present new approaches. It points out differences between analysis of the whole somatotype and the separate components. In the future other methods will probably emerge and receive acceptance in the area of somatotyping. Finally, other tools should be used in conjunction with somatotyping to answer detailed questions regarding physique and composition.

****

PART 3: EQUATIONS FOR SOMATOTYPE ANALYSIS

The following equations are used in calculation and analysis of somatotype data. Items 1 and 2 are for calculating and plotting the somatotype. Items 3-6 are for analysis of the whole somatotype. Item 7 suggests methods for comparisons by components.

1. The anthropometric somatotype.

Endomorphy = - 0.7182 + 0.1451 (X) - 0.00068 (X^2) + 0.0000014 (X^3)

Mesomorphy = (0.858 HB + 0.601 FB +0.188 CAG + 0.161 CCG) - (0.131 H) + 4.5

Ectomorphy:
If HWR ^{3} 40.75, then Ectomorphy = 0.732 HWR - 28.58
If HWR < 40.75 and > 38.25, then Ectomorphy = 0.463 HWR - 17.63
If HWR £ 38.25, then Ectomorphy = 0.1 (or recorded as ½)

Where: X = (sum of triceps, subscapular and supraspinale skinfolds) multiplied by (170.18/height in cm); HB = humerus breadth; FB = femur breadth; CAG = corrected arm girth; CCG = corrected calf girth; H = height; HWR = height / cube root of weight.
CAG and CCG are the girths corrected for the triceps or calf skinfolds respectively as follows: CAG = flexed arm girth - triceps skinfold/10; CCG = maximal calf girth - calf skinfold/10.

2. Plotting somatotypes on the 2-D somatochart.

X-coordinate = ectomorphy - endomorphy

Y-coordinate = 2 x mesomorphy - (endomorphy + ectomorphy)

3. Somatotype categories.

The following seven categories can be used for simplified classification of samples if the numbers are large enough. These categories can be used in Chi-square analyses. (Thirteen categories are defined in Carter, 1980, 1996, and Carter and Heath, 1990.) (See Part 1 for 4 and 13 categories.)

Central: no component differs by more than one unit from the other two.

Endomorph: endomorphy is dominant, mesomorphy and ectomorphy are more than one-half unit lower.

Endomorph-mesomorph: endomorphy and mesomorphy are equal (or do not differ by more than one-half unit), and ectomorphy is smaller.

Mesomorph: mesomorphy is dominant, endomorphy and ectomorphy are more than one-half unit lower.

Mesomorph-ectomorph: mesomorphy and ectomorphy are equal (or do not differ by more than one-half unit), and endomorphy is smaller.

Ectomorph: ectomorphy is dominant, endomorphy and mesomorphy are more than one-half unit lower.

Ectomorph-endomorph: endomorphy and ectomorphy are equal (or do not differ by more than one-half unit), and mesomorphy is lower.

4. Somatotype attitudinal distance (SAD).

The SAD is the exact difference, in component units between two somatotypes (A, an individual or group; B, an individual or group), or between two somatotype group means (e.g. A and B), or between a subject and a group (e.g. subject A and group B).

$$\mathbf{SAD_{A;B} = \sqrt{[(ENDO_A - ENDO_B)^2 + (MESO_A - MESO_B)^2 + (ECTO_A - ECTO_B)^2]}}$$

Where: ENDO = endomorphy; MESO = mesomorphy; ECTO = ectomorphy

The somatotype attitudinal mean (SAM) is the mean of a group of somatotypes.

$$\mathbf{SAM = \sum SAD_i / n_X}$$

Where: SAD_i = somatotype of each subject minus the mean somatotype of the group; n_X is the number in the group x.

The somatotype attitudinal variance (SAV) is the variance of the group.

$$\mathbf{SAV = \sum SAD_i^2 / n_X}$$

The standard deviation of the somatotypes about SAM is $\sqrt{SAV}$

5. Longitudinal somatotype ratings.

The same subject is remeasured over time; calculated by the migratory distance (MD). The MD is the sum of the SADs, calculated from each consecutive pair of somatotypes of the subject.

MD (a;z) = SAD (a;b) + SAD (b;c) + ... + SAD (y;z)

Where: a = first rating; b = second rating; y and z = last two ratings.

6. Comparisons between independent samples.

(1). T-RATIO:

$$\mathbf{t = \bar{S}_1 - \bar{S}_2 / \sqrt{[\sum (SAD_1^2) + \sum (SAD_2^2) / (n1 + n2 - 2) * (1/n1 + 1/n2)]}}$$

Where: $\bar{S}_1$ = mean of group 1, $\bar{S}_2$ = mean of group 2; SAD_1 and n1, and SAD_2 and n2, refer to groups 1 and 2 respectively.

(2) ANOVA , F-RATIO:

$$\mathbf{F = (SS_t / df_t) / (SS_e / df_e) = MS_t / MS_e}$$

$$\mathbf{SS_t = \sum n_j (\bar{S}_j - M_o)^2 \text{, and } SS_e = \sum \sum (SAD_j^2)}$$

$$\mathbf{SS_{t1} = n1 (\bar{S}_1 - M_o)^2 \text{, and } SS_{t2} = n2 (\bar{S}_2 - M_o)^2}$$

Where: Subscripts - t = treatment; e = error; j = j groups; M_o = overall mean somatotype for combined groups;

For a worked examples see Carter et al. (1983, p. 205). For an alternative SAD-ANOVA, see Cressie et al. (1986).

7. Comparisons by components.

(1) To identify the contributions of the three-somatotype components to possible differences between samples, use standard MANOVA designs or Discriminant Analysis.

(2) For quantitative analysis of the longitudinal change in somatotype components use MANOVA techniques with a within-subject design.

(3) To assess the relationship over time for a component use interage partial correlations for one component while holding the other two constant, i.e. $r_{1,23}$.

Computer programs

Somatotype – Calculation and Analysis V1.1. Monte Goulding, Sweat Technologies, Mitchell Park, South Australia. A new comprehensive user-friendly PDF platform program designed to calculate individual and group somatotypes, import somatotype data from, and export to, other files. Reports show statistics, somatoplots, categories, and comparisons of individuals and/or groups. The software is available as a Single User License (Price ~ US$49.95) or a Site License (Price ~ US$495.00). Download a 5 hour free trial version from: www.sweattechnologies.com/somatotype. (Email: monte@sweattechnologies.com.)

HC_StypeCalculation-03.xls. A simple program for calculating the Heath-Carter somatotype from anthropometry. Useful for individual or group data. No plotting or analysis.

LIFESIZE. V1.0 for Windows (or MAC), (1999), by **T. Olds and K. Norton**, is an anthropometric profiling and analysis program. It is a tool which assists in the collection and analysis of anthropometric data, has a database containing norms for somatotype, body fat and skinfolds for the general population and specific sporting groups. It is an interactive and exploratory educational tool for students in anthropometry. (Available from Human Kinetics.)

B.O.R.I.S. (2002). Pedro Alexander. A CD-Rom software program in Spanish for anthropometric evaluation, including somatotype. Available from Dr. Miguel Albaran, Puerto Rico. Email: umetma@yahoo.com

References

Bell, W. (1993). Body size and shape: a longitudinal investigation of active and sedentary boys during adolescence. Journal of Sports Science, 11:127-138.

Beunen, G.; Claessens, A.; Lefevre, J.; Ostyn, M.; Renson, R.; Simon, J. (1987). Somatotype as related to age at peak velocity and peak velocity in height, weight and static strength in boys. Human Biology, 59:641-655.

Cameron, N. (1984). The Measurement of Human Growth. Beckenham: Croon Helm Ltd.

Carter, J.E.L. (1980). The Heath-Carter Somatotype Method, 3rd edition. San Diego: San Diego State University Syllabus Service.

Carter, L. (1996). Somatotyping. In: K. Norton and T. Olds (Eds.), Anthropometrica, Chapt. 6, pp. 147-170. Sydney: University of New South Wales Press.

Carter, J.E.L., & Heath, B.H. (1990). Somatotyping - Development and Applications. Cambridge: Cambridge University Press.

Carter, J.E.L., Ross, W.D., Duquet, W., & Aubry, S.P. (1983). Advances in somatotype methodology and analysis. Yearbook of Physical Anthropology, 26, 193-213.

Carter, J.E.L.; Duquet, W.; Rempel, R. Letter to the Editor, Response. Am. J. Hum. Biol. 10:2-4, 1998.

Carter, J.E.L.; Duquet, W. Letter to the Editor, Response. American Journal of Human Biology, 11:434-436, 1999.

Cressie, N.A.C. Letter to the Editor. American Journal of Human Biology, 10:1-2, 1998.

Cressie, N.A.C. Letter to the Editor. American Journal of Human Biology, 11:433-434, 1999.

Cressie, N.A.C., Withers, R.T. and Craig, N.P. (1986). Statistical analysis of somatotype data. Yearbook of Physical Anthropology, 29, 197-208.

Duquet, W. & Carter, J.E.L. (2001). Somatotyping. In: R. Eston & T. Reilly (Eds.), Kinanthropometry and Exercise Physiology Laboratory Manual: Tests, procedures and data. Vol. 1, Anthropometry, Chapt. 2. London: E & F.N. Spon.

Heath, B.H., & Carter, J.E.L. (1967). A modified somatotype method. American Journal of Physical Anthropology, 27, 57-74.

Hebbelinck, M.; Duquet, W. (1977). Applications of the somatotype attitudinal distance to the study of group and individual somatotype status and relations. In Eiben OG (ed): Growth and Development: Physique, pp. 377-384. Budapest: Akademiai Kiado (Hungarian Academy of Sciences).

Hebbelinck, M.; Duquet, W.; Borms, J.; Carter, J.E.L. (1995). Stability of somatotypes: a longitudinal growth study in Belgian children followed from 6 to 17 years. American Journal of Human Biology, 7: 575-588.

Hebbelinck, M.; Duquet, W.; Ross, W.D. (1973). A practical outline for the Heath-Carter somatotyping method applied to children. In Pediatric Work Physiology Proceedings, 4th International Symposium, 71-84. Wingate Institute, Israel.

ISAK (2001). International Standards for Anthropometric Assessment. Underdale, S.A.; International Society for the Advancement of Kinanthropometry.

Norton, K. & Olds, T. (1996). Anthropometrica. Sydney: University of New South Wales Press.

Rempel, R. (1994). A modified somatotype assessment methodology. M.Sc. Thesis, Simon Fraser University, Burnaby, Canada.

Rosique, J.; Rebato, E.; Gonzalez Apraiz, A.; Pacheco, J.L. (1994). Somatotype related to centripetal fat patterning of 8- to 19-year-old Basque boys and girls. American Journal of Human Biology, 6:171-181.

Ross, W.D. & Marfell-Jones, M.J. (1991). Kinanthropometry. In J.D. MacDougall, H.A. Wenger & H.J. Green (Eds.), Physiological Testing of the High-Performance Athlete (pp. 223-308). Champaign, IL: Human Kinetics.

Ross, W.D., Carr, R.V. & Carter, J.E.L. (1999). Anthropometry Illustrated (CD-Rom). Surrey: Turnpike Electronic Publications, Inc.

Schmidt, P.K. & Carter, J.E.L. (1990). Static and dynamic differences among five types of skinfold calipers. Human Biology, 62, 369-388.

Sodhi, H.S. (1991). Sports Anthropometry (A Kinanthropometric Approach). Mohali: ANOVA Publications.

Winer, B.J. (1971). Statistical Principles in Experimental Design. New York: McGraw-Hill.

Figure legends

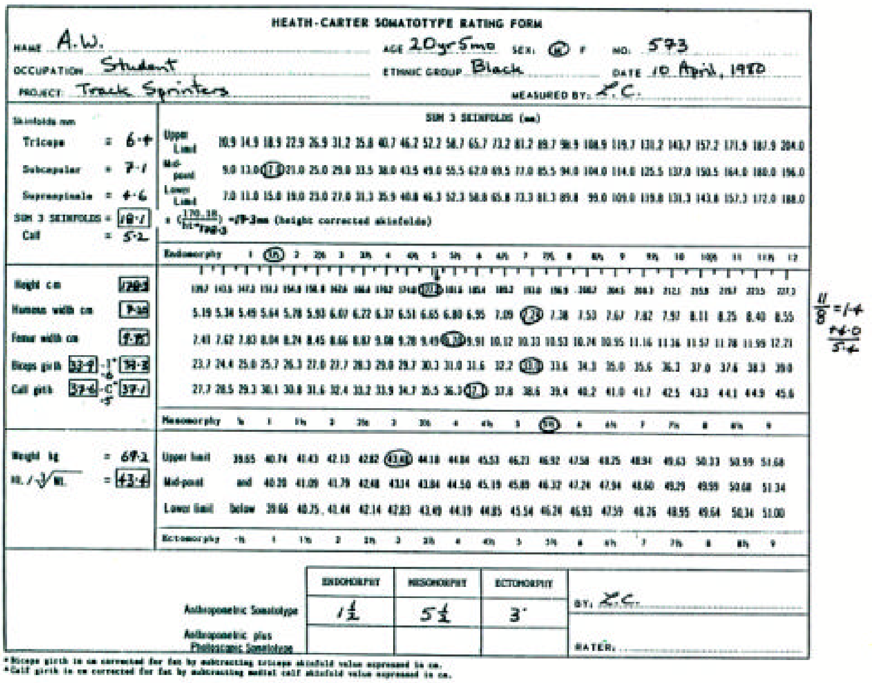

Figure 1. Calculations of the anthropometric somatotype for subject 573 using the rating form.

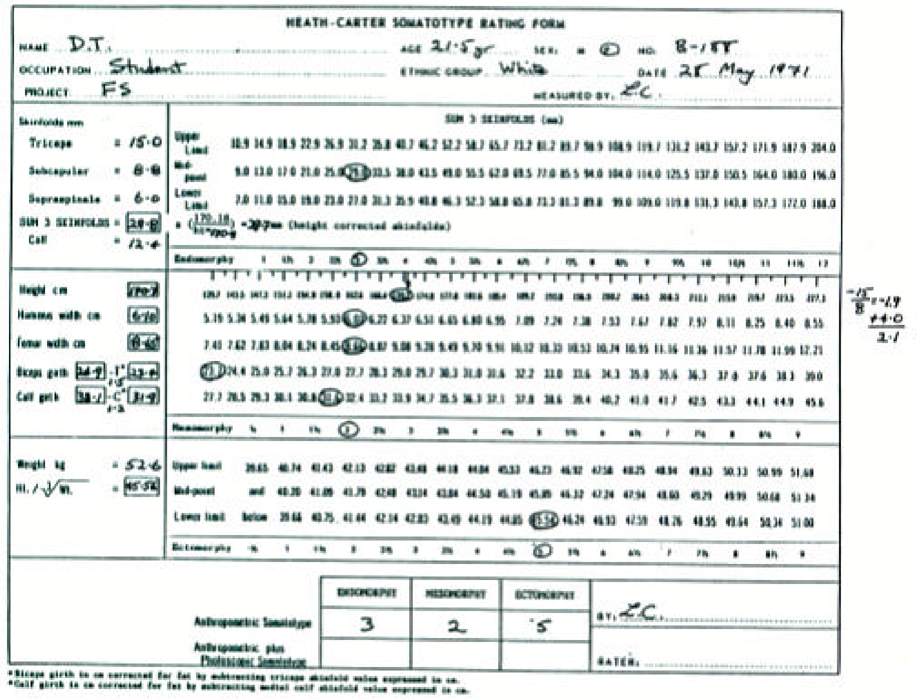

Figure 2. Calculations of the anthropometric somatotype for subject B-188 using the rating form.

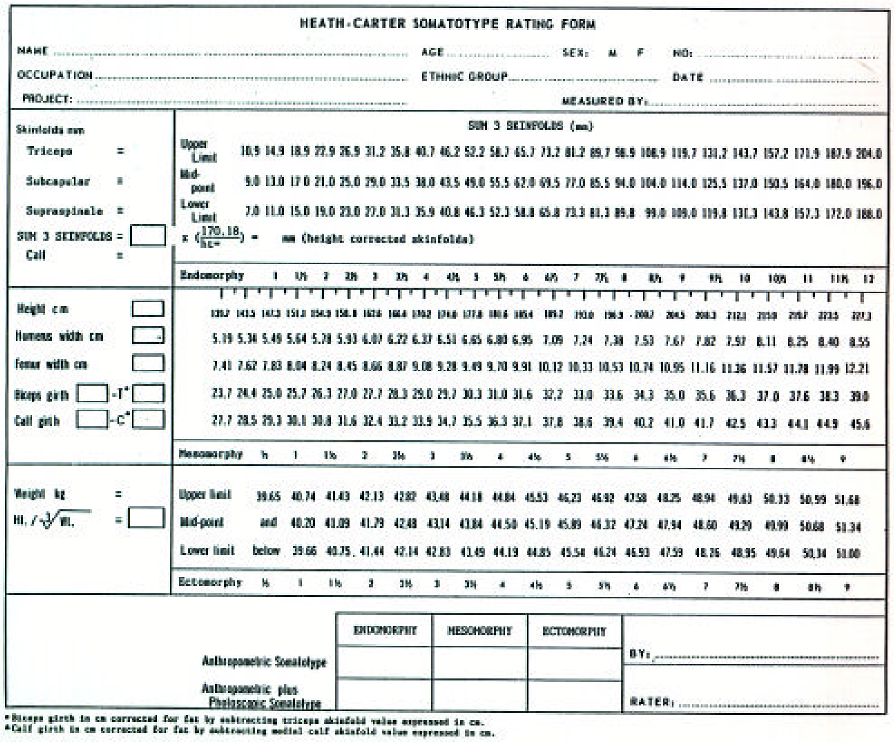

Figure 3. Blank somatotype rating form.

A	½ - 1	1	2	3	4	5	6	7	8	9	B
15.40										119,	50.91
15.20									118,	129, 219	50.25
15.00								117,	128, 218		49.59
14.80								127, 217	138, 318<br>228,		48.93
14.60							126, 216	137, 317<br>227,			48.27
14.40							136, 316<br>226,	237, 327			47.61
14.20						135, 315<br>225,	146, 416<br>236, 326				46.95
14.00					134, 314<br>224,	145, 415<br>235, 325	246, 426<br>336,				46.28
13.80					144, 414<br>234, 324	245, 425<br>335,					45.62
13.60				233,	154, 514<br>244, 424<br>334,	255, 525<br>345, 435					44.96
13.40				153, 513<br>333,	254, 524<br>344, 434						44.30
13.20			242, 422	163, 613<br>253, 523<br>343, 433	354, 534<br>444,						43.64
13.00			162, 612<br>252, 522	263, 623<br>353, 533<br>443,							42.98
12.80		341, 431	172, 712<br>262, 622<br>352, 532<br>442,	363, 633<br>453, 543							42.32
12.60		171, 711<br>261, 621<br>351, 531<br>441,	182, 812<br>272, 722<br>362, 632<br>452, 542								41.66
12.40		181, 811<br>271, 721<br>361, 631<br>451, 541	282, 822<br>372, 732<br>462, 642<br>552,								40.99
12.20		191, 911<br>281, 821<br>371, 731<br>461, 641<br>551,									40.33
12.00		291, 921<br>381, 831<br>471, 741<br>561, 651									39.67
11.70	5-6½-½, 6-6-1<br>10-2-1, 2-10-1	391, 931<br>481, 841<br>571, 751									38.68
11.40	10-3-1, 3-10-1<br>10½-2½-½, 11-2-1	491, 941<br>581, 851<br>671, 761									37.69
11.00	6½-7½-½, 7-7-1<br>10½-3½-½, 11-3-1	4-10-1, 10-4-1<br>5-9-1, 9-5-1<br>6-8-1, 8-6-1									36.37
10.50	7-8-1, 8-7-1<br>11½-3½-½, 12-3-1	4-11-1, 11-4-1<br>5-10-1, 10-5-1<br>6-9-1, 9-6-1									34.71
10.00	7½-8½-½, 8-8-1<br>11½-4½-½, 12-4-1<br>13-3-1	5-11-1, 11-5-1<br>6-10-1, 10-6-1<br>7-9-1, 9-7-1									33.06
9.50	12-5-1<br>13-4-1	7-10-1, 10-7-1<br>8-9-1, 9-8-1<br>11-6-1									31.41
9.00	12-6-1<br>13-5-1<br>14-4-1	8-10-1, 10-8-1<br>9-9-1<br>11-7-1									29.75

A = height in inches / cube root of weight in pounds
B = height in centimeters / cube root of mass in kilograms

Figure 4. Distribution of somatotypes according to the HWR (height/weight$^{1/3}$).

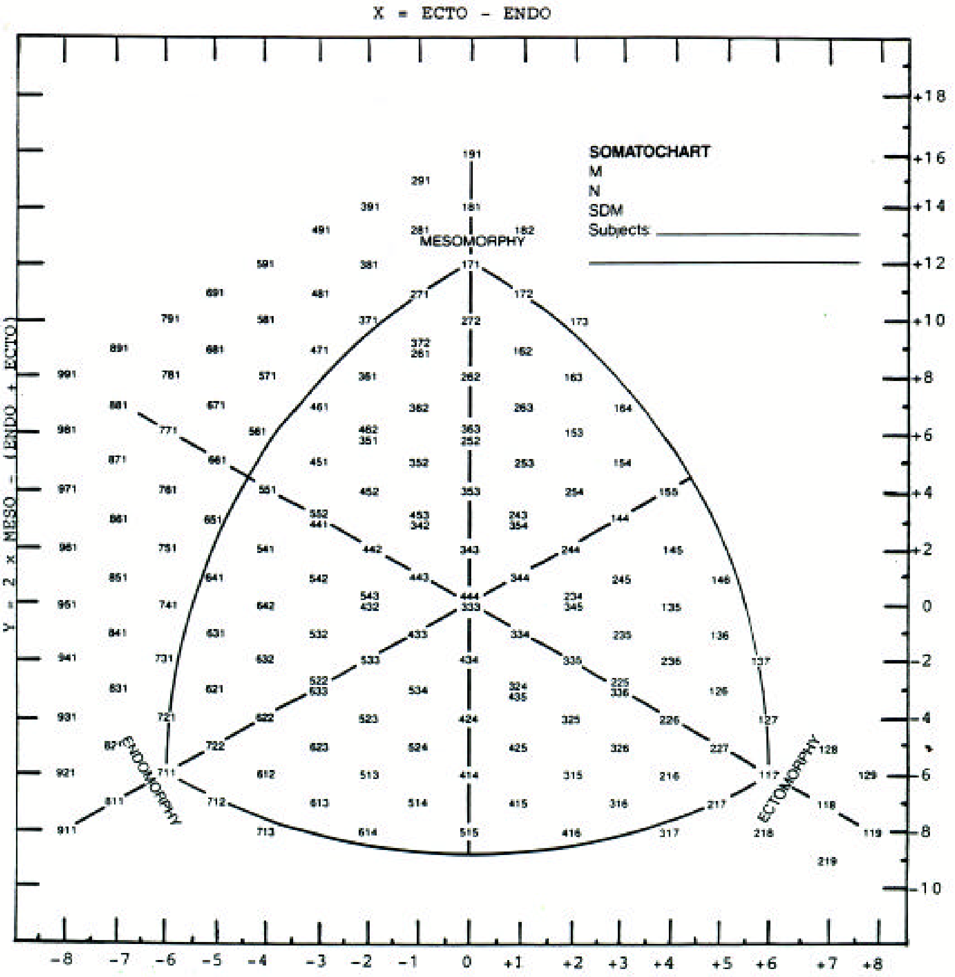

Figure 5. The 2-D somatochart and X,Y coordinates.

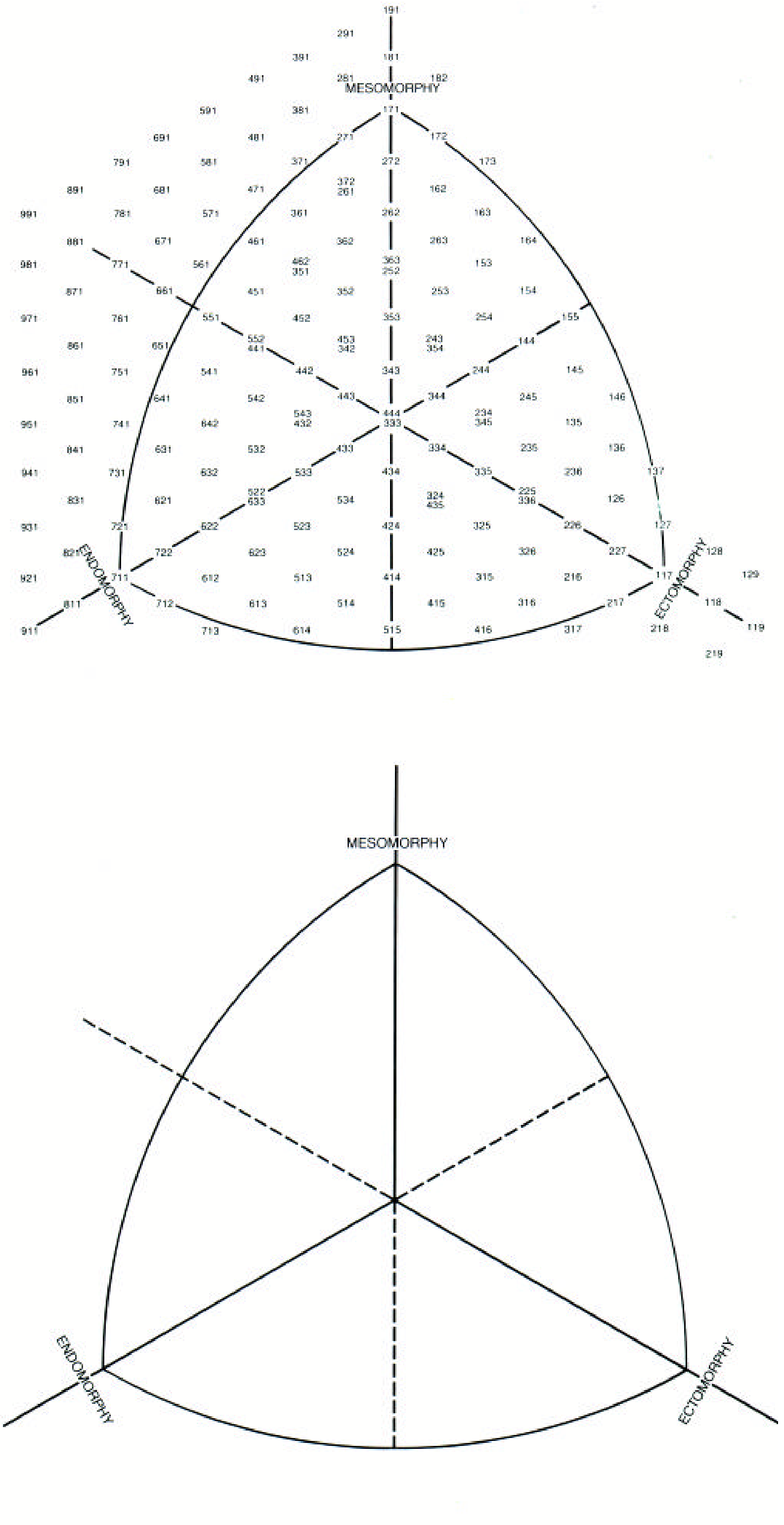

Figure 6 a,b Blank somatocharts.

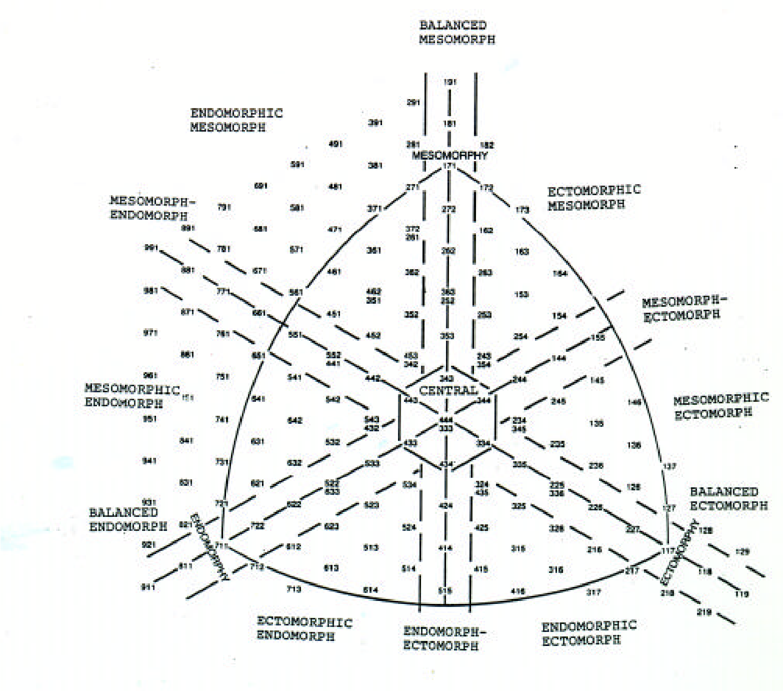

Figure 7. Somatotype categories labeled according to Carter and Heath (1990). Somatoplots falling within the same area are grouped by category.
